# GRP-YOLOv5: An Improved Bearing Defect Detection Algorithm Based on YOLOv5

**DOI:** 10.3390/s23177437

**Published:** 2023-08-26

**Authors:** Yue Zhao, Bolun Chen, Bushi Liu, Cuiying Yu, Ling Wang, Shanshan Wang

**Affiliations:** 1 Faculty of Computer and Software Engineering, Huaiyin Institute of Technology, Huaian 223003, China; 2 Department of Physics, University of Fribourg, CH-1700 Fribourg, Switzerland

**Keywords:** bearing defect detection, YOLOv5, gamma transformation, C2f structure, PConv convolution

## Abstract

Currently, most chemical transmission equipment relies on bearings to support rotating shafts and to transmit power. However, bearing defects can lead to a series of failures in the equipment, resulting in reduced production efficiency. To prevent such occurrences, this paper proposes an improved bearing defect detection algorithm based on YOLOv5. Firstly, to mitigate the influence of the similarity between bearing defects and non-defective regions on the detection performance, gamma transformation is introduced in the preprocessing stage of the model to adjust the image’s grayscale and contrast. Secondly, to better capture the details and semantic information of the defects, this approach incorporates the ResC2Net model with a residual-like structure during the feature-extraction stage, enabling more nonlinear transformations and channel interaction operations so as to enhance the model’s perception and representation capabilities of the defect targets. Additionally, PConv convolution is added in the feature fusion part to increase the network depth and better capture the detailed information of defects while maintaining time complexity. The experimental results demonstrate that the GRP-YOLOv5 model achieves a mAP@0.5 of 93.5%, a mAP@0.5:0.95 of 52.7%, and has a model size of 25 MB. Compared to other experimental models, GRP-YOLOv5 exhibits excellent performance in bearing defect detection accuracy. However, the model’s FPS (frames per second) performance is not satisfactory. Despite its small size of 25 MB, the processing speed is relatively slow, which may have some impact on real-time or high-throughput applications. This limitation should be considered in future research and in the optimization efforts to improve the overall performance of the model.

## 1. Introduction

Bearings are important components in chemical equipment that support rotating shafts. If the bearing performance is poor, it can lead to imbalance in the rotating parts, causing equipment vibration, increased noise, and even equipment failure, shutdown, or damage, thereby affecting the stability and reliability of the equipment [1]. Additionally, bearing failures can result in the detachment of rotating parts from the equipment, leading to mechanical hazards and potentially causing serious accidents, thereby resulting in personal injury to workers and property damage. Maintenance requires equipment downtime, which also reduces production efficiency. Therefore, bearings play a crucial role in chemical production. Various factors affect the quality of bearings, with one of the major factors being the occurrence of defects such as grooves, abrasions, and scratches on the bearing’s outer surface during production, assembly, and transportation. These defects pose safety risks to the normal operation of supporting equipment. Therefore, defect detection in bearings is of paramount importance.

With technological advancements, modern detection methods such as computer vision and artificial intelligence have been gradually applied to the surface-defect detection of bearings. These methods offer advantages such as high intelligence, accuracy, and efficiency. Both two-stage object detection algorithms like R-CNN [2], Fast R-CNN [3], and Faster R-CNN [4], as well as single-stage object detection algorithms [5] like YOLOv5, have been widely used in defect detection. Single-stage object detection algorithms, in particular, have demonstrated superior performance as they directly perform sorting and regression from feature maps, thus eliminating the need for candidate box generation and achieving faster detection speed with significant advantages in accuracy. However, when facing the diverse types, shapes, and sizes of defects in bearing defect detection, as well as the interference from background noise and other factors affecting defect target images, traditional single-stage algorithms often struggle to achieve satisfactory detection results.

Therefore, this paper proposes an accurate and real-time bearing defect detection algorithm based on the single-stage YOLOv5 model. The specific contributions of this paper are as follows:

- In the preprocessing stage, a gamma transformation preprocessing technique is introduced to perform a nonlinear transformation of the image’s grayscale values, thus enhancing the image’s contrast and brightness. This process eliminates the similarity between bearing defects and non-defective regions, reducing the occurrence of false positives and false negatives.
- In the backbone network, a novel ResC2Net residual-like network structure is proposed to enhance the network’s feature representation and attention enhancement capabilities, further improving the detection performance for small bearing defects.
- In the model’s neck, a PConv local convolution operation is added to improve the accuracy of defect edge detection without increasing the computational complexity.

## 2. Research Status at Home and Abroad

### 2.1. Traditional Detection Methods

Defect detection methods have been a long-standing research area of interest in both engineering and academia. Traditional defect-detection methods encompass technologies such as signal processing, image processing, feature extraction, and machine learning. Zhao et al. [6] provided a comprehensive overview of recent research in defect-detection systems based on wireless sensors from different perspectives of sensing principles, wireless networks, and power sources. They reviewed conventional rail inspection techniques, including vibration, ultrasonic, electromagnetic, and visual detection; in addition, they compared their performances. Zhang et al. [7] proposed an improved active noise reduction-based rail defect detection method using acoustic emission technology, thereby focusing on noise interference suppression in high-speed defect detection. By introducing multi-stage noise elimination that combined SANC and ANC, as well as tongue-shaped curves with exponential adjustment factors, the performance of the variable step-size algorithm was enhanced. Mandriota et al. [8] compared three methods—Gabor filters, wavelets, and Gabor wavelet filters—for defect detection based on the surface texture analysis of the tracks. However, compared to general object detection, defect detection may involve issues such as limited data and intra/inter-class variance. Due to the limited availability of defect data, particularly for specific defect types, sample sizes might be limited. This can lead to data scarcity challenges during model training and testing, potentially impacting detection accuracy and generalization ability. Additionally, defects encompass diverse types and may exhibit different properties in varying locations and environments. This presents challenges related to intra-class and inter-class variance. Defect detection requires addressing the similarity within intra-class samples and the differences between inter-class samples to ensure accurate identification and localization of the different defect types. Therefore, compared to general object detection, defect detection faces more formidable challenges. The purpose of this paper is to explore research methodologies in the field of bearing defect detection. For instance, Liu et al. [9] designed and studied a system for measuring inner race cone angles and detecting surface defects in bearings. The system utilized an industrial camera for image acquisition and the intelligent detection of inner race cone angles and surface defects through image processing, thereby aiming to improve bearing quality. F et al. [10] proposed a new adaptive segmentation threshold selection algorithm, and they evaluated a large number of published sequential search algorithms to select the optimal feature subset for classification. They also compared their computational requirements, eventually providing the classification results for 540 defect images. Ng [11] used the technology of illumination with a circular light source to detect bearing defects and conducted quality testing on ball bearings. Chen et al. [12]. proposed a method based on image registration technology to eliminate the false magnetic indications in magnetic particle flaw detection results. They also introduced the Hough transform into defect recognition in magnetic particle flaw detection, which exhibited robustness against point noise in the magnetic particle flaw detection results. Li et al. [13] presented a classification method based on fiber optic sensing technology by analyzing the detection mechanism of Y-shaped and coaxial fiber optic sensors, as well as established mathematical models. They conducted numerical simulations using the MATLAB software and studied the influence of fiber optic parameters on the modulation characteristics. Ma et al. [14] proposed a dynamic threshold algorithm for defect detection based on variations in ball bearing parameters according to certain standards. This algorithm combined the entire feature value with local feature values by utilizing the difference between the measured values of the normal surface and the defect surface, as well as demonstrated the distribution characteristics of measured values for qualified and defective ball bearings through the data distribution of probability density functions. Esmaeil et al. [15] used a multi-probe FDDP (frequency domain differential probe) device, based on the impact of different spectra, to detect surface cracking faults on the outer wall of rolling bearings. This method enabled a fast and accurate detection of crack locations and sizes, but it requires specialized equipment and high-precision data analysis. Gu et al. [16] proposed a machine vision-based automatic detection and recognition method for bearing surface defects, which improved the integrity and accuracy of bearing surface defect segmentation by applying the gamma correction algorithm to the original image and adaptively selected the improved Canny algorithm based on iterative threshold segmentation and the Otsu algorithm. Ma et al. [17] effectively identified different colors by using a CCD industrial color camera to capture real-time images in the production line and connected the same pixel area based on Halcon, thus eliminating interference from the bearings around the wires through image segmentation, which eventually led to completing the partition program and classifying the defect area.

It can be observed that traditional detection methods generally suffer from issues such as low efficiency, low accuracy, and significant human intervention. Moreover, they fail to meet the requirements of modern industrial production for efficient and accurate detection.

### 2.2. Deep Learning-Based Detection Methods

Currently, methods based on convolutional neural networks (CNNs) have been widely applied in the field of bearing defect detection. These methods leverage their powerful feature extraction and representation capabilities for high-dimensional data, and have achieved success in various detection tasks [18]. Zhao et al. [19] proposed a two-stage network-based method for detecting surface defects on bearings. It significantly improved the detection accuracy by removing the influence of oil droplets on the defects. This method introduces the SENet attention mechanism and CBAM attention module to restore detail and improve texture details in the oil droplet removal regions. Xu et al. [20] proposed a deep learning-based approach for identifying fault defects in road subsurfaces through using Ground Penetrating Radar (GPR) data. The Faster R-CNN framework was employed and, specific to road subsurface defects, the method integrated feature fusion, the Adversarial Spatial Dropout Network (ASDN), soft NMS (Non-Maximum Suppression), and data augmentation as improvement strategies through which to enhance the recognition accuracy. Ma et al. [21] proposed a novel classification method for surface defects in bearings through using an integrated framework of transfer learning convolutional neural networks (CNNs), thus enabling efficient training and enhanced feature extraction capabilities under small-scale datasets and multiple lighting conditions. Tao et al. [22] presented a new AI-based PIMLM labeling algorithm and designed the You-Only-Look-Once-OurNet (YOLO-OurNet) deep learning network based on a single-stage architecture for detecting defects on the rolling surface of roller cones. Zheng et al. [23] replaced the traditional DarkNet-53 with BNA-Net to obtain more information about large-scale feature maps and to extract more features from the small-sized defects. Additionally, they proposed attention prediction subnets and defect localization subnets to extract deep structural semantic information, as well as to enhance the detection of large-scale targets. Song et al. [24] improved YOLOv4 model by introducing a focal loss through which to address the imbalanced information between bearing defect samples and the background. Wen et al. [25] combined traditional computer vision methods with deep learning, and they used an improved RetinaNet model to achieve surface defect detection on bearing rollers. Xie et al. [26] proposed a bearing roller end-face defect detection algorithm based on an improved YOLOv5n model. They used photometric stereo vision to reconstruct the three-dimensional shape of the surface and addressed surface deformation issues using an improved Frankot–Chellappa integral algorithm. The DenseFuse network was used to fuse gamma-corrected images and curvature maps, generating a dataset that combines the advantages of both images by enhancing defect features and improving the accuracy of object detection. Yuan et al. [27] proposed an improved automatic detection region preprocessing and a multi-head self-attention module in the transformer to enhance the feature extraction capability for medium-sized surface defects. Moreover, they improved the model’s convergence speed and detection accuracy by using instance normalization instead of batch normalization.

Although deep learning-based object detection algorithms have achieved some results in bearing defect detection, issues such as false positives, false negatives, and detection challenges with overlapping and small defects remain.

## 3. Algorithm Description

**Figure 1:**
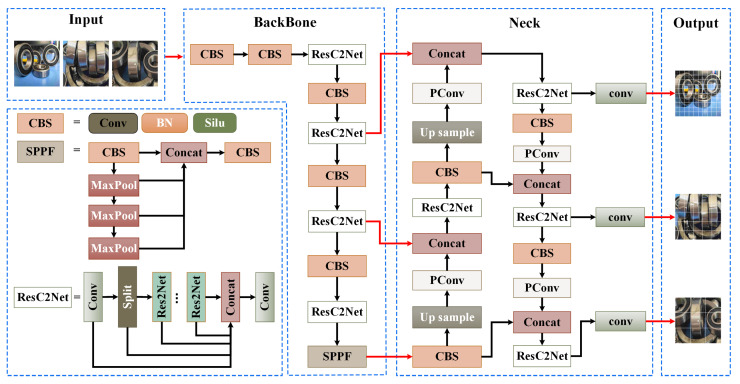
Structural Diagram of the GRP-YOLOv5 Bearing Defect Detection Model.

As a consumable component, bearings are prone to various damage during production and transportation, resulting in a wide range of defect types. This diversity makes it challenging to capture the details and deep semantic information of defects during the detection process. To address this issue, this paper proposes an improved bearing defect detection model. Figure 1 illustrates the overall structure of the proposed GRP-YOLOv5 bearing defect detection model.

The model first employs gamma transformation to adjust the brightness levels of the pixels within the bearing defect detection region, thereby reducing the similarity between defect target areas and the background. Additionally, this paper introduces a network model called ResC2Net, which is based on the C2f [28], and Res2Net structures [29]. It enhances the model’s nonlinear expression capacity through inter-layer residual-like structures, and it enables the learning of deeper semantic information. Furthermore, in the feature fusion section, a lightweight PConv convolution [30] was added to enhance the model’s ability to handle bearing defect edge information.

### 3.1. Data Preprocessing

The experimental data used for training the network model in this paper are from a chemical equipment bearing defects dataset. It includes various common bearing defects such as grooves, abrasions, and scratches. To enhance the model’s generalization ability and robustness, random scaling, image flipping, and Mosaic Augmentation techniques were applied to augment the original data, thereby expanding the dataset and increasing its diversity. Data augmentation techniques not only effectively prevent overfitting, improve the model’s generalization ability, and stability, but they also help the model better understand the manifestation of bearing defects in different scenarios, thereby facilitating better recognition and detection of these defects, as well as improving the industrial production efficiency and quality.

Furthermore, this paper employs gamma transformation as an automatic technique through which to adjust the brightness and contrast of the images, thereby reducing background noise in the defect images and enhancing the model’s robustness. Gamma transformation is a nonlinear transformation used to adjust the brightness and contrast of images. It is based on the nonlinear characteristics of human visual perceptions of brightness, and it achieves brightness adjustment by altering the pixel value mapping relationship in the image. The transformation formula is given as follows:

(1) $$I^{\prime }=c*I^{\gamma }$$

where *I* is the pixel value in the original image, $I^{\prime }$ is the pixel value after gamma transformation, γ is the gamma value used for brightness and contrast adjustment, and *c* is a constant used to scale the transformed pixel values within the possible range of the pixel values.

**Figure 2:**
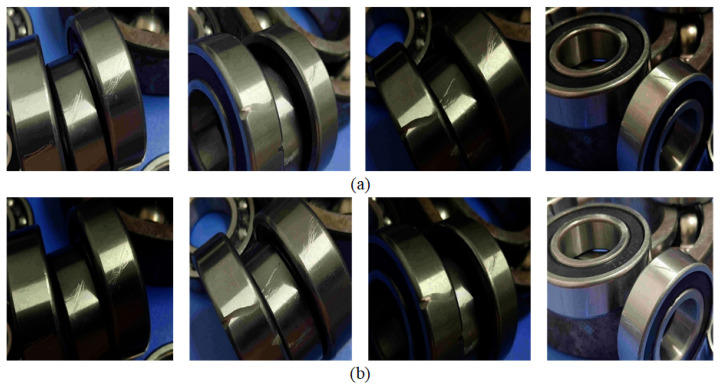
Illustration of the Effect of Random Gamma Transformation. (**a**) Original Image. (**b**) Random Gamma-Transformed Image.

In this paper, when applying gamma transformation, the pixel values of the image are first normalized to the range of 0 to 1 by dividing each pixel value by the maximum possible value. Then, the normalized pixel values are raised to the power of the gamma value, which makes darker pixel values brighter and brighter pixel values darker. Finally, the transformed pixel values were rescaled to the original range of pixel values by multiplying them by the maximum possible value. Figure 2 illustrates the images after gamma transformation in this algorithm.

From the observation of Figure 2, it can be noticed that the gamma transformation adaptively adjusts the overall brightness of the image. In certain regions where defects are present and affected by glare, reducing the contrast and brightness is necessary to clearly display these defects, which helps reduce noise and smooth the image. However, due to the overall darkness of some parts of the image, certain defects may not be clearly visible. In such cases, increasing the contrast and brightness can effectively aid in displaying these defects.

### 3.2. Backbone

**Figure 3:**
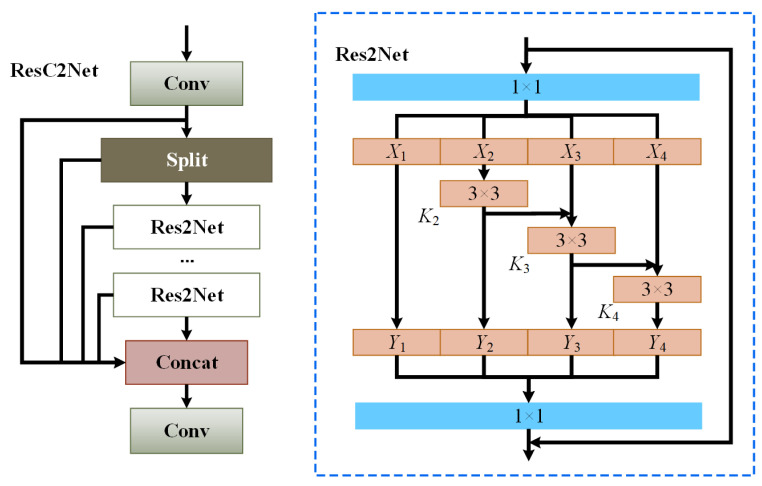
Illustration of the Overall Structure of ResC2Net.

In the backbone network, the conventional YOLOv5 network model utilizes CSPDarknet53 for feature extraction. In CSPDarknet53, the C3 structure serves as a module for constructing the feature extraction layers. It simply concatenates the feature maps processed by bottleneck modules with the original input feature map. However, this straightforward fusion approach may not fully capture the correlations among the different feature maps regarding the defects, resulting in suboptimal fusion effects. Additionally, the bottleneck structure in the C3 module is formed by sequentially stacking convolutional layers, which limits the receptive field of each layer’s feature map and hinders the handling of multi-scale defect targets. Therefore, we propose a ResC2Net structure, as shown in Figure 3.

In response to the challenge of insufficiently fused defect information, the C2f structure is introduced as a module aimed at enhancing feature representation and boosting network performance. The C2f structure partitions the input feature map into two segments, processes them through two branches, and then merges the outcomes of these branches so as to amplify feature representation and to facilitate the flow of information across diverse branches, thereby bolstering the fusion effect of the bearing defect features.

Additionally, to overcome the limitation of confined receptive fields, the Res2Net structure was introduced. The Res2Net module can inherently assimilate information from all of the preceding subsets of the feature maps; it can also extract the varying scales and dimensions of receptive fields, as well as enhance the efficacy of feature extraction through parallel connections and information amalgamation. By substituting grouped convolutions for the 3×3 convolutional layers and interconnecting diverse groups layer by layer, the hierarchical intra-group connections—resembling residual connections—enable a continuous alteration of receptive fields. This empowers the model to capture local and global bearing defect features at a more intricate level.

The ResC2Net design effectively harnesses the benefits of C2f and Res2Net to adeptly capture the features at different scales and semantic strata. The C2f structure employs bottleneck blocks for feature extraction, encompassing multiple convolutional layers and activation functions, to amplify the network’s capacity for nonlinear expression. However, the bottleneck structure, being composed of stacked convolutional layers, restricts the receptive fields of the feature maps in each layer. As a remedy, this paper substitutes the bottleneck structure with the Res2Net configuration, enhancing each layer’s feature map receptive fields through 3×3 convolutions and inter-group residual connections to accommodate defects of varying magnitudes. The information exchange, facilitated by C2f, engenders adequate interactions between the features within different branches, thus enhancing the amalgamation of local and global information. Concurrently, the multi-scale feature representation proficiency of Res2Net empowers the model with a more intricate comprehension of features across various scales, thereby elevating the model’s grasp of image intricacies and structures. Notably, while achieving potent feature extraction, ResC2Net keeps parameter count and computational overhead in check. Through lightweight convolution operations and ingenious parameter sharing mechanisms, the model judiciously employs computational resources, thereby maintaining modest computational costs. This endows the model with efficiency when handling expansive datasets and resource-constrained scenarios.

Given the ability of the ResC2Net module to extract more comprehensive feature representations through interactions with varying scales of convolutional kernels and channels, it excels in capturing defects of diverse dimensions and intricacies. This architectural approach equips the model to perform adeptly across distinct defect types and severities, thus substantiating its robust generalization capabilities.

The overall structure flow of ResC2Net involves the input tensor passing through a 1×1 convolutional layer. The output is then split into two tensors, with one of them serving as input to multiple Res2Net modules. Each Res2Net module processes the subset of $x_{i}\left(i\in 2,\ldots ,s\right)$ from the previous layer’s output through a 3×3 convolutional layer $K_{i}$. The subset $x_{i}\left(i\in 3,\ldots ,s\right)$ is then added to the output of $K_{i-1}$ and jointly inputted into $K_{i}$ for processing. The output of each Res2Net module, denoted as $y_{i}$, is calculated according to Equation (2).

(2) $$y_{i}=\left\{\begin{array}{cc} x_{i} & i=1\\ K_{i}\left(x_{i}\right) & i=2\\ K_{i}\left(x_{i}+y_{i-1}\right) & 2<i\leq s \end{array}\right.$$

The output tensors $y_{i}$ from each Res2Net module are sequentially concatenated with another tensor, resulting in a series of output tensors. These output tensors can be viewed as partial feature representations from each Res2Net module. These partial feature representations are concatenated in order to form a larger feature tensor. Finally, this feature tensor is processed by a 1×1 convolutional layer to obtain the final output tensor.

### 3.3. Neck

**Figure 4:**
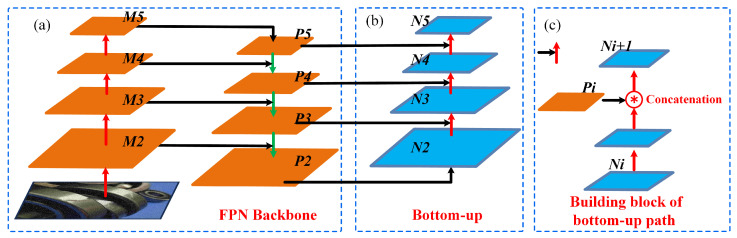
Path Aggregation Network (PAN) Structure. (**a**) FPN backbone. (**b**) The bottom-up structure of the Path Aggregation Network. (**c**) Building block of the bottom-up path.

**Figure 5:**
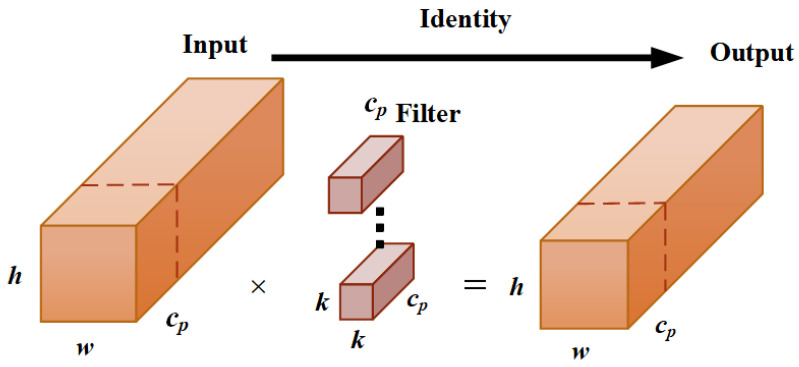
The schematic diagram of the PConv local convolution.

In the neck network, we adopted the Path Aggregation Network (PAN) [31] for feature fusion. Compared to the Feature Pyramid Network (FPN) [32] shown in Figure 4a, PANet introduces bottom-up pathways (as shown in Figure 4b) to gradually aggregate and integrate the defect features of different scales, thus enabling the network to provide a more comprehensive and rich representation of the defect features. The PANet structure is illustrated as below:

The input image size in the study is 640×640×3. After multiple convolutional downsampling operations, different-sized defect feature maps M2, M3, M4, and M5 are extracted for constructing the axle defect feature fusion network. The feature map *M*5 is adjusted in the channel number using a 1×1 convolution, thus resulting in the feature map P5. P5 is then upsampled by a factor of 2 and fused with M4 to obtain the feature layer P4. Similarly, P4 is upsampled by a factor of 2 and fused with M3 to obtain the feature layer P3. Furthermore, P3 is upsampled by a factor of 2 and fused with M2, and feature extraction is performed using the Cross Stage Partial (CSP) local network, thus yielding the feature map P2. After adjusting the channel number of P2, the feature map N2 is obtained. The bottom-up process (as shown in Figure 4c) is then applied to integrate *N*2 with P3, P4, and P5, resulting in the feature maps N3, N4, and N5. By combining the interaction information between the defect feature maps from both top-down and bottom-up processes, the final updated axle defect feature map is generated. However, in PANet, traditional convolutional operations may lead to information loss when dealing with detailed features, such as axle defects. To address this issue, we introduced the PConv local convolution operation in the neck module (as shown in Figure 5), which enables the model to accurately capture the edge, texture, and shape features of the defects, thereby facilitating a better fusion of the detailed information in the axle defect regions, as well as improving the detection accuracy and robustness of the axle defects.

During the PConv local convolution, only a portion of the input channels undergo spatial feature extraction via convolution; meanwhile, the remaining channels are left unchanged. In the training process, the input feature map is divided into two parts: the convolutional output obtained from the partial convolution operation on the front portion of channels and the remaining portion of the channels. These two parts of the channels are then concatenated along the channel dimension to form the final output feature map.

### 3.4. Loss Function

The loss function is used to measure the difference between the predicted results and the ground truth labels, thus guiding the training process of the model. The loss function used in the model is defined as Equation (3), where the total loss $L_{loss}$ is the sum of the confidence loss $l_{obj}$, classification loss $l_{cls}$, and regression loss $l_{box}$.

(3) $$L_{\mathrm{loss}}=\lambda_{\alpha }l_{obj}+\lambda_{\beta }l_{cls}+\lambda_{\delta }l_{box}$$

Here, $\lambda_{\alpha }$, $\lambda_{\beta }$, and $\lambda_{\delta }$ are the balance coefficients.

The confidence loss measures the difference between the predicted box confidence and the ground truth labels. The classification loss measures the difference between the predicted class probabilities and the ground truth labels. Both the confidence loss and the classification loss are calculated using the binary cross-entropy loss function, as expressed in Equations (4) and (5), respectively:

(4) $$l_{obj}=-\sum_{i=1}^{N}{\hat{p}}_{i}log\left(p_{i}\right)+\left(1-{\hat{p}}_{i}\right)log\left(1-p_{i}\right)$$

(5) $$l_{cls}=-\sum_{i=1}^{N}\hat{y_{i}}log\left(y_{i}\right)+\left(1-{\hat{y}}_{i}\right)log\left(1-y_{i}\right)$$

Here, *N* is the number of predicted boxes, and $p_{i}$ represents the ground truth confidence indicating the presence of an object at that location. ${\hat{p}}_{i}$ is the predicted confidence value output by the network after applying the sigmoid function, thus representing the predicted probability of the object’s presence. $y_{i}$ is the ground truth class indicating the object’s true category. ${\hat{y}}_{i}$ is the predicted class distribution output by the network after applying the softmax function.

The regression loss measures the difference between the predicted boxes and the ground truth boxes. The regression loss is calculated using the CIOU_Loss, as shown in Equations (6)–(9):

(6) $${CIOU}_{-}Loss\;=1-CIOU$$

(7) $$CIOU=IOU-\frac{\rho^{2}}{c^{2}}-\alpha v$$

(8) $$v=\frac{4}{\pi^{2}}{\left(arctan\frac{w_{l}}{h_{l}}-arctan\frac{w_{p}}{h_{p}}\right)}^{2}$$

(9) $$\alpha =\frac{v}{1-IOU+v}$$

Here, ρ represents the Euclidean distance between the predicted box and the ground truth box’s center points. *c* is the normalization factor of the diagonal length of the target box. α is a balancing parameter used to balance the position and size errors of the predicted box. *v* is a correction factor used to reduce the overlap differences between the large and small objects. $w_{l}$ and $h_{l}$ are the width and height of the ground truth box, respectively. $w_{p}$ and $h_{p}$ are the width and height of the predicted box, respectively.

The loss function serves as the objective function for the optimization algorithm. By minimizing the loss function, the model’s parameters are adjusted to better fit the training data, thereby improving the model’s performance.

## 4. Experimental Results and Analysis

### 4.1. Dataset Introduction

**Figure 6:**
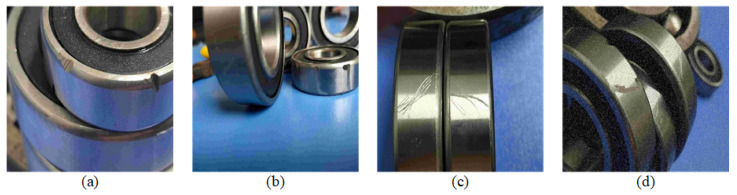
The various types of defects in the dataset. (**a**) Groove defect. (**b**) Abrasion defect. (**c**) Scratch defect. (**d**) All defects.

In this paper, the defect detection data for the bearings in the chemical plant equipment before usage were collected. The dataset comprises a total of 6543 images [33], which were divided into training, validation, and testing sets at an 8:1:1 ratio, respectively. Prior to conducting experiments, the images in the dataset were annotated with labeling. Upon completion of the annotations, the corresponding text files were generated for each image. These text files contain information about the location and category of the various defects present in the images, enabling us to use them as input data during model training, validation, and testing. This dataset takes into account the diversity and uncertainty of the bearing defects in various stages such as production, assembly, and transportation, which encompassed different types of defects including groove defects, scratch defects, and abrasion defects. These varying defect types have differing impacts on bearing performance during operation, necessitating accurate defect detection and classification in practical applications. The dataset’s bearing defects also exhibited varying shapes, sizes, and positions. This requires defect detection algorithms to possess strong generalization capabilities and robustness to handle a range of defect shapes and positions that may arise in real-world scenarios. Figure 6 showcases partial example images of the different defect types present in the dataset.

### 4.2. Evaluation Metrics

The performance of the model in this paper was evaluated using two metrics: detection accuracy and model size. In terms of detection accuracy, metrics such as recall(R), precision(P), average precision (AP), and mean average precision (mAP) were utilized for evaluation purposes. Recall refers to the proportion of the detected targets to the total number of targets, and it can be calculated using the following formula:

(10) $$Recall=\frac{TP}{TP+FN}$$

Here, TP represents true positives (correctly detected positive samples), and FN represents false negatives (positive samples not detected).

Precision measures the proportion of true positives among the detected targets and can be calculated as follows:

(11) $$Precision=\frac{TP}{TP+FP}$$

Here, TP represents true positives, and FP represents false positives (negative samples mistakenly detected as positive).

AP represents the average precision value at different recall levels, and it can be calculated using the following formula:

(12) $$AP=\sum_{i=1}^{n}P\left(R\right)dR$$

Here, P(R) represents the precision at a specific recall level *R*, and dR denotes the increment of the recall. Precision and recall are often computed at different confidence thresholds, and a precision–recall curve is then plotted. Next, the area under the curve (average precision) is calculated to measure the performance.

mAP denotes the average of AP values for different classes, and it can be calculated using the following formula:

(13) $$mAP=\frac{\sum_{i=1}^{n}P\left(R\right)dR}{n}$$

Here, *n* represents the number of classes.

FNR (False Negative Rate) is the proportion of actual positives that are incorrectly predicted as negatives. It can be calculated using the following formula:

(14) $$FNR=\frac{FN}{TP+FN}$$

where FN stands for false negative, representing the number of samples that are actually positive (true positives) but are incorrectly classified as negative (false negatives), and TP stands for true positive, representing the number of samples that are actually positive (true positives) and are correctly classified.

F-Score is a commonly used performance metric for classification models as it balances the precision and recall metrics. It is computed based on the true positive and false positive counts from model predictions, as well as by the correct capture of true positives. The formula to calculate F-Score is as follows:

(15) $$F-Score=2\times \frac{Precision\;\times \;Recall}{Precision\;+\;Recall\;}$$

where Precision represents the proportion of samples predicted as positives that are actually positives, and Recall represents the proportion of all true positives correctly predicted by the model. F-Score ranges between 0 and 1, with values closer to 1 indicating a better model performance.

In this paper, in addition to assessing detection accuracy, various metrics such as Model Size, GFLOPs, and FPS were employed to evaluate the performance and efficiency of the deep learning models. These metrics aid in comprehending aspects like model complexity, computational load, and inference speed. Within the experimental section of this study, a comprehensive explanation of the experimental methodology and obtained results is provided, which is followed by a thorough discussion and analyses of the models.

### 4.3. Experimental Setup

All experiments in this study were conducted on a Windows 10 operating system. The training process utilized a GPU device, specifically a Tesla V100-SXM2-16GB with a memory capacity of 16130 MiB. The experiments were conducted using Python version 3.8, with the PyTorch 1.8.0 framework and CUDA version 10.2.89. The model was trained for 200 iterations, with a batch size of 16. During the training process, the SGD optimizer with a momentum of 0.937 was employed for optimization. The learning rate was adjusted periodically with a Warm-Up strategy, where the initial learning rate was set to 0.01 and the learning rate decay weight was set to 0.0005. In the Warm-Up phase, the learning rate was updated using a one-dimensional linear interpolation for each iteration until it reached 0.002. Additionally, a Cosine Annealing learning rate decay method was utilized to automatically adjust the learning rate.

### 4.4. Analysis of Defect Detection Results

#### 4.4.1. Ablation Experiment

To investigate the contributions of the improved modules in the algorithm, ablation experiments were conducted in this study. Independent experiments were performed on the dataset to evaluate the three proposed improvements while keeping the experimental conditions consistent, including the devices used, training hyperparameters, and iteration counts. These experiments aimed to reveal the roles of the different components in the object detection task, as well as provided valuable insights into the performance enhancement and improvement of the object detection algorithm. The experimental results are presented in Table 1.

In this study, a series of improvements were applied to the YOLOv5 model, including the incorporation of gamma transformation, the ResC2Net module, and PConv convolution. Based on the ablation experimental results (as presented in Table 1), compared to the YOLOv5s model, the individual introduction of gamma transformation, the ResC2Net module, and PConv convolution led to respective enhancements of 0.8%, 1.1%, and 1.9% in terms of recall. Regarding precision, when compared to the YOLOv5s model, the individual inclusion of Gamma transformation and PConv convolution resulted in reductions of 0.4% and 1.2%, respectively, while the addition of the ResC2Net module brought about an increase of 1.1%. By employing the False Negative Rate (FNR) to evaluate whether positive instances were missed during detection, the results showed that, when contrasted with the YOLOv5s model, the separate incorporation of gamma transformation, the ResC2Net module, and PConv convolution reduced the false negative rates by 0.8%, 1.1%, and 1.9%, respectively. The comprehensive *F-Score*, which considers both precision and recall, demonstrated that introducing gamma transformation, the ResC2Net module, and PConv convolution individually resulted in *F-Score* improvements of 0.2%, 1.1%, and 0.4%, respectively, when compared to the YOLOv5s model. For the mAP@0.5 metric, introducing gamma transformation, the ResC2Net module, and PConv convolution separately led to enhancements of 0.8%, 1.9%, and 0.9%, respectively, when compared to the YOLOv5s model. However, when considering the broader range of mAP@0.5:0.95, the introduction of gamma transformation caused a reduction of 0.1%, while the incorporation of the ResC2Net module and PConv convolution resulted in increments of 1.6% and 0.8%, respectively. These experimental findings underscore the effectiveness of these three enhancement methods. Notably, introducing gamma transformation alone resulted in decreased Precision and mAP@0.5:0.95 values, while the introduction of PConv convolution alone led to reduced Precision. Therefore, in future work, further research into the influencing factors of gamma transformation and PConv convolution is warranted, along with attempts to optimize these methods to enhance their stability in various scenarios. It is worth noting that simultaneously incorporating gamma transformation, the ResC2Net structure, and PConv convolution into the YOLOv5 model resulted in a 2.2% improvement in recall, a 3.7% improvement in precision, a 2.8% improvement in mAP@0.5, a 2.1% improvement in mAP@0.5:0.95, and a 2.9% improvement in *F-Score*. These outcomes further validate the stabilizing and performance-enhancing effects of introducing gamma transformation and these two modules to the model.

#### 4.4.2. Visualization Result Analysis

**Figure 7:**
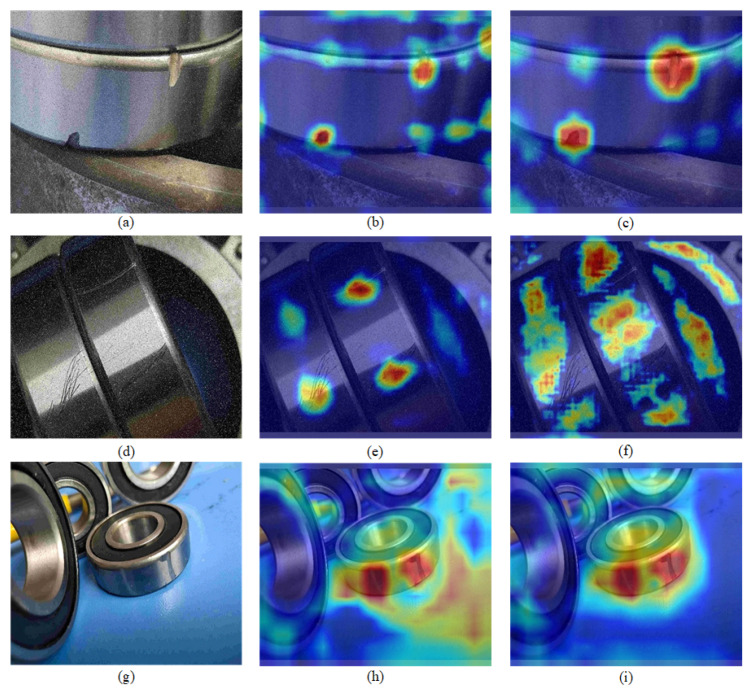
Heat maps of the Defect Types in the Dataset. (**a**) Original Image of the Groove Defect. (**b**) Heat map of the Groove Defect in YOLOv5. (**c**) Heat map of the Groove Defect in GRP-YOLOv5. (**d**) Original Image of the Abrasion Defect. (**e**) Heat map of the Abrasion Defect in YOLOv5. (**f**) Heat map of the Abrasion Defect in GRP-YOLOv5. (**g**) Original Image of the Scratched Defect. (**h**) Heat map of the Scratch Defect in YOLOv5. (**i**) Heat map of the Scratch Defect in GRP-YOLOv5.

In this study, heat maps were utilized to visualize the results of defect detection. By observing the highlighted regions in the heat maps, the detection capability of the model and the accuracy of target localization can be intuitively evaluated. The experimental results are shown in Figure 7.

When comparing the defect heat maps of the YOLOv5 model and the GRP-YOLOv5 model in Figure 7, it is evident that the GRP-YOLOv5 model exhibits a more pronounced focus on the defect targets; in addition, it demonstrates accurate localization and recognition of the defect objects. This indicates that the GRP-YOLOv5 model effectively captures the crucial features for bearing defect detection tasks, thus enabling precise bounding box predictions.

#### 4.4.3. Model Training

In this study, no pre-trained models were used, and all models were trained from scratch. The loss curves after training are shown in the following figure. Based on the line chart analysis, the loss values of the YOLOv5 model and the GRP-YOLOv5 model in the object detection task were compared.

**Figure 8:**
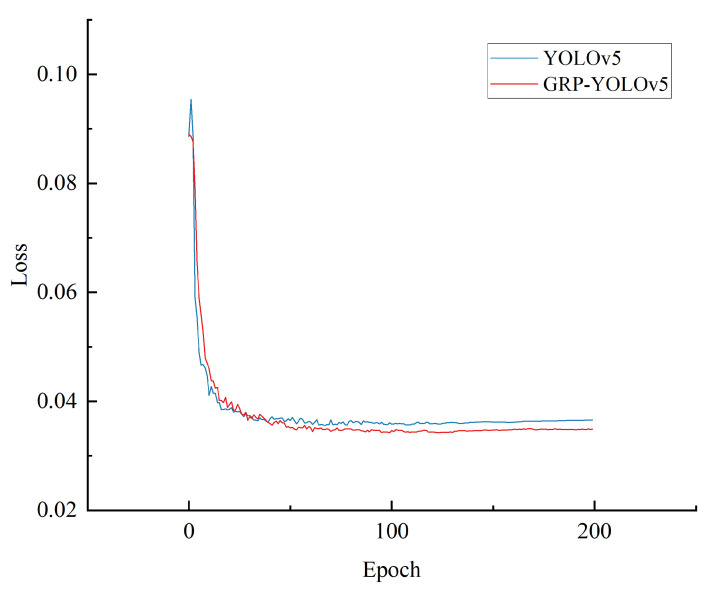
Comparison of the Model Loss Functions.

From Figure 8, it can be observed that the GRP-YOLOv5 model demonstrated lower loss values, indicating a better performance in object detection. The lower loss values suggest that the GRP-YOLOv5 model can more accurately locate and classify the defective objects, thus improving the accuracy of object detection. Moreover, the loss function curve indicates that the model has achieved a certain level of performance, and that further training may no longer yield significant benefits.

#### 4.4.4. Detection Performance for Different Types of Defects

**Figure 9:**
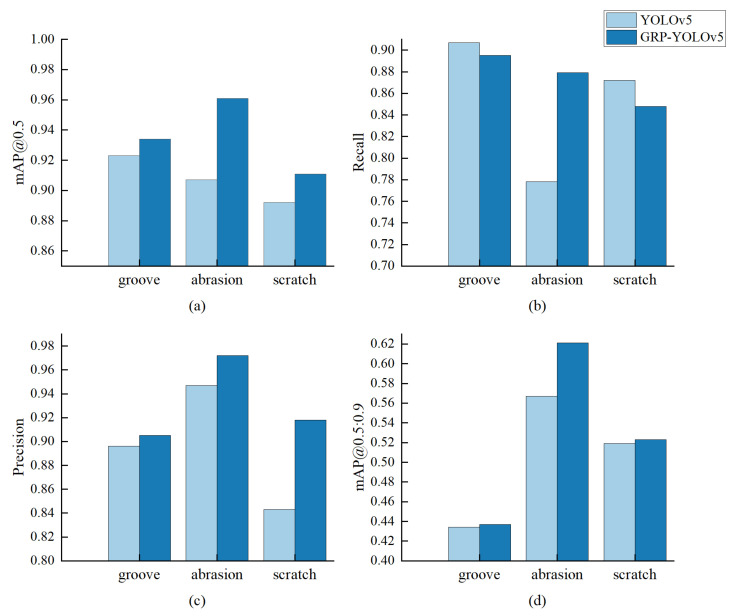
Defect Detection Results. (**a**) Illustration of mAP@0.5 for different defects. (**b**) Illustration of recall for different defects. (**c**) Illustration of precision for different defects. (**d**) Illustration of mAP@0.5:0.95 for different defects.

In this study, the performance of the GRP-YOLOv5 model was evaluated on the common types of bearing defects, including grooves, abrasions, and scratches. Evaluation metrics such as precision, average precision (mAP) at different thresholds (mAP@0.5, mAP@0.5:0.95), and recall were used to assess the performance of the GRP-YOLOv5 model, which were then compared with the traditional YOLOv5 model.The results are shown in Figure 9.

The experimental results demonstrate that the GRP-YOLOv5 model outperforms the traditional YOLOv5 model in terms of precision, mAP@0.5, and mAP@0.5:0.95 for groove, scratch, and dent defect detection. This indicates that the GRP-YOLOv5 model exhibits a higher accuracy and overall performance in localizing and classifying defects. However, in terms of recall, the traditional YOLOv5 model performs better than the GRP-YOLOv5 model for groove and scratch defects. Analysis revealed that the GRP-YOLOv5 model shows a significant increase in recall for scratch defects compared to the YOLOv5 model. The diversity and varying difficulty of defect categories can result in differences in shape, texture, and color, thereby making scratch defects more easily detectable while the other two categories may pose more challenges. These factors can affect the model’s discrimination ability and recall for different targets.

#### 4.4.5. Test Results Visualization

**Figure 10:**
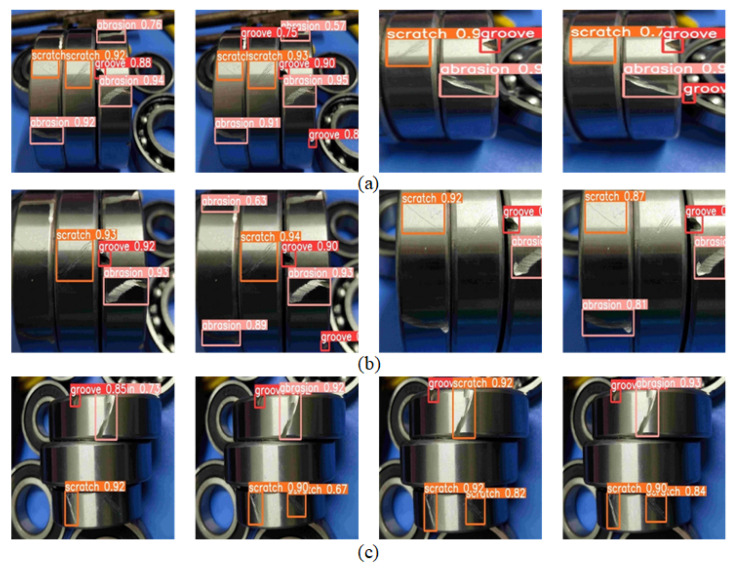
Test Results Visualization. (**a**) Groove defect detection image. (**b**) Abrasion defect detection image. (**c**) Scratch defect detection image. The first, second, third, and fourth images from left to right in (**a**–**c**) respectively represent the testing results of YOLOV5, GPR-YOLOv5, YOLOV5, and GPR-YOLOv5.

This experiment aimed to evaluate the performance of the GRP-YOLOv5 model on the test set for different defect categories, including groove, scratch, and dent defects. The test results are presented in Figure 10, showcasing the detection bounding box outputs of the YOLOv5 model and the GRP-YOLOv5 model for the three defect categories.

The test set prediction results are shown in Figure 10. It provides a performance comparison and analysis between the two models for different defect categories. The YOLOv5 model exhibits shortcomings in perceiving grayscale and fine details, resulting in missed detections for groove defects that are located in concealed and dark areas. In contrast, the GRP-YOLOv5 model improves the detection results of groove defects by enhancing its capability to capture details in dark regions through the introduction of gamma transformation and the ResC2Net module. Regarding scratch defect detection, the YOLOv5 model demonstrates inaccurate extractions of shape and texture features, leading to missed detections. On the other hand, the GRP-YOLOv5 model improves its ability to capture details of scratch defects by incorporating non-linear transformations and channel interactions. In terms of dent defect detection, since scratches and dents share similarities, the YOLOv5 model exhibits false positives. The GRP-YOLOv5 model, with better detail capture and feature fusion, enhances the accuracy of dent defect detection and reduces false positive cases.

## 5. Comparative Experiments

In the Section 5, a comprehensive comparative analysis was performed involving the GRP-YOLOv5 algorithm and the six widely recognized object detection algorithms: SSD, RetinaNet [34], YOLOv6s6 [35], YOLOv7 [36], YOLOv8s, and YOLOv5s. Additionally, several defect detection algorithms were selected as benchmark references, including Improved Faster R-CNN [4,37] and Improved YOLOxs [33,38]. The conducted comparative experiments were based on uniform experimental settings and a dataset, thereby employing standard evaluation metrics like mean average precision (mAP), model size, GFLOPs, FPS, and others so as to comprehensively evaluate the performance of the diverse algorithms in the context of bearing defect detection.The experimental results are shown in Table 2.

The experimental results show that our GRP-YOLOv5s model achieves outstanding performance—with mAP@0.5 and mAP@0.5:0.95 scores of 93.5% and 52.7%, respectively—when compared to the commonly used object detection algorithms, thus surpassing them. Additionally, the GRP-YOLOv5s model demonstrates superiority in terms of model size and GFLOPs, with values of 25.17 MB and 16.0 GFLOPs, respectively. However, the FPS of the GRP-YOLOv5s is relatively lower at 68.5 f/s. In comparison to defect detection algorithms, GRP-YOLOv5 performs the best with an mAP score of 93.5%, which is 11.3% higher than Improved Faster R-CNN’s 82.2% and 3.8% higher than Improved YOLOxs’s 89.7%. For the mAP@0.5:0.95 metric, GRP-YOLOv5 also outperforms other algorithms, achieving 52.7%, indicating its high accuracy even under stricter confidence thresholds. The GRP-YOLOv5 model boasts the smallest model size with only 25.17 MB. Moreover, in terms of computational complexity (GFLOPs), GRP-YOLOv5 excels, with only 16.0 GFLOPs, compared to the 941.0 GFLOPs required for Improved Faster R-CNN and the 26.7 GFLOPs required for Improved YOLOxs. Therefore, GRP-YOLOv5 exhibits a higher computational efficiency. Regarding the FPS f/s metric, GRP-YOLOv5 achieves 68.5 f/s, which is higher than the 18.3 f/s of Improved Faster R-CNN and the 50.4 f/s of Improved YOLOxs.

## 6. Conclusions

This study aims to address the challenges in the field of bearing defect detection by proposing an enhanced YOLOv5 network architecture for defect detection. The algorithm introduces gamma transformation, a ResC2Net residual-like structure, and PConv local convolution to adjust image grayscale and contrast, thus enhancing the model’s perception and representation of target objects while resolving issues of information loss in bearing defect detection. The experimental results demonstrate that the GRP-YOLOv5 algorithm achieves remarkable performance with mAP@0.5 and mAP@0.5:0.95 scores of 93.5% and 52.7%, respectively, thus surpassing other detection models. Furthermore, the GRP-YOLOv5 model excels in terms of model size and GFLOPs, which were measured at 25.17 MB and 16.0 GFLOPs, respectively. However, the FPS of GRP-YOLOv5s was relatively lower at 68.5 f/s. The overall research outcomes highlighted significant performance advantages of the GRP-YOLOv5 algorithm in object detection tasks, particularly in it showcasing impressive results under high-precision requirements. In future work, we will continue optimizing the model to enhance its frame rate performance in real-time scenarios. Additionally, we plan to validate the model’s generalization capability on different datasets, and to explore various hardware acceleration techniques in order to achieve more efficient inference speeds.

## Figures and Tables

**Table 1:** Ablation Experiment Results.

Algorithm	G	R	P	Recall	Precision	mAP@0.5	mAP@0.5:0.95	FNR	*F-Score*
YOLOv5s				85.2%	89.5%	90.7%	50.6%	14.8%	87.3%
YOLOv5s+G	*√*			86.0%	89.1%	91.5%	50.5%	14.0%	87.5%
YOLOv5s+R		*√*		86.3%	90.6%	92.6%	52.2%	13.7%	88.4%
YOLOv5s+P			*√*	87.1%	88.3%	91.6%	51.4%	12.9%	87.7%
GRP-YOLOv5	*√*	*√*	*√*	87.4%	93.2%	93.5%	52.7%	12.6%	90.2%

In the table, “G” represents gamma transformation, “R” represents the ResC2Net module, and “P” represents the PConv convolution.

**Table 2:** Comparative Experiments of the Different Models.

Algorithm	mAP@0.5	mAP@0.5:0.95	Model Size	GFLOPs	FPS
SSD	72.3%	36.9%	90.84 MB	62.7	104.8 f/s
RetinaNet	91.9%	51.4%	138.16 MB	146.0	26.8 f/s
YOLOv6s6	88.2%	48.4%	-	45.2	30.4 f/s
YOLOv7	93.2%	52.5%	141.38 MB	105.1	51.3 f/s
YOLOv8s	90.8%	52.4%	42.29 MB	28.4	357.1 f/s
YOLOv5s	90.7%	50.6%	26.74 MB	15.8	77.5 f/s
Improved Faster R-CNN	82.2%	41.2%	107.55 MB	941.0	18.3 f/s
Improved YOLOxs	89.7%	51.7%	33.91 MB	26.7	50.4 f/s
GRP-YOLOv5	93.5%	52.7%	25.17 MB	16.0	68.5 f/s

## Data Availability

Not applicable.

## Sample Availability

Samples of the codes are available from the authors.
